# Protective Effects of Sal B on Oxidative Stress-Induced Aging by Regulating the Keap1/Nrf2 Signaling Pathway in Zebrafish

**DOI:** 10.3390/molecules26175239

**Published:** 2021-08-29

**Authors:** Erzhuo Li, Yunhao Wang, Qiao Li, Li Li, Lijun Wei

**Affiliations:** 1 State Key Laboratory of Urban Water Resource and Environment, Harbin Institute of Technology, Harbin 150090, China; 1182800217@stu.hit.edu.cn; 2 School of Life Science and Technology, Harbin Instituted of Technology, Harbin 150001, China; wangyunhao666@sina.com (Y.W.); 17B928007@stu.hit.edu.cn (Q.L.)

**Keywords:** salvianolic acid B, zebrafish, aging, oxidative stress

## Abstract

The models of oxidative damage-induced aging were established by adding ethanol (C_2_H_5_OH), hydrogen peroxide (H_2_O_2_) and 6-hydroxydopamine (6-OHDA) to zebrafish embryos in this research. To find effective protective drugs/foods, Salvianolic acid B (Sal B) was added after the embryos were treated by these oxidative reagents. After being treated with ethanol, H_2_O_2_ and 6-OHDA, the morphological changes were obvious and the deformities included spinal curvature, heart bleeding, liver bleeding, yolk sac deformity and pericardial edema, and the expression of oxidative stress-related genes *Nrf2b*, *sod1* and *sod2* and aging-related genes *myl2a* and *selenbp1* were significantly up-regulated compared to the control group. While after adding 0.05 μg/mL and 0.5 μg/mL Sal B to the ethanol-treated group, death rates and MDA levels decreased, the activity of antioxidant enzyme (SOD, CAT and GSH-Px) changed and *Nrf2b*, *sod1*, *sod2*, *myl2a*, *selenbp1*, *p53* and *p21* were down-regulated compared to the ethanol-treated group. The bioinformatics analysis also showed that oxidative stress-related factors were associated with a variety of cellular functions and physiological pathways. In conclusion, Sal B can protect against aging through regulating the Keap1/Nrf2 pathway as well as antioxidative genes and enzyme activity.

## 1. Introduction

Senescence is a spontaneous and inevitable process of organisms with the passage of time, manifesting as a decline in function, adaptability and immunity [1]. With the increase in the proportion of the elderly population in the total population, a variety of diseases of the elderly also came along, and the delay of aging and the study of diseases associated with aging are attracting more and more attention. Among these are neurodegenerative diseases. Neurodegenerative diseases tend to occur in the elderly population, with the characteristics of slow disease development, high incidence and low cure rate, which have a great impact on the healthy life of patients. Oxidative stress is an important pathogenesis of such diseases [2]. The mechanisms of cellular senescence include telomere shortening, DNA damage accumulation, abnormal oncogene activation, metabolic changes and high levels of production of reactive oxygen species (ROS) [3].

Among many model animals, we chose to use zebrafish to construct the aging model. The zebrafish (*Danio rerio*) gene sequence is up to 70–80% homologous with human, and has the characteristics of small size, strong reproductive ability and a transparent body of embryos and juveniles [2]. Through the study of zebrafish, it was found that as they grow older, the phenomenon of spinal curvature might be caused by abnormal muscle conditions, which is consistent with human characteristics in morphology. Various data showed that during the aging process of zebrafish, the activity of senescence-related gene beta galactosidase increased in the skin, oxidative protein accumulated in the muscle and lipofuscin content increased significantly in the liver, which is consistent with the reported aging characteristics of humans and mice [4,5]. At present, zebrafish has been widely used as a model animal in the research fields of developmental science, genetics, behavioral science and molecular biology [6,7]. More and more applications have been made in the study of neurological diseases such as Parkinson’s disease [2]. Doctor Maiquan Li of Zhejiang university used the zebrafish model of 6-hydroxydopamine (6-OHDA) neurotoxin-induced oxidative metabolism of dopamine to study the effect of phenethyl ethanol glycoside on the prevention of Parkinson’s syndrome [2]. Liu jiao, Ph.D. of Nanjing Medical University, used 27-hydroxyl cholesterol to influence neural behavior and senescence in young zebrafish [8]. Li hongyan research group of Qingdao ocean university studied zebrafish and proposed that CA2, MYL2A, MYL2B and SELENBP1 could be used as molecular markers of senescence [9].

Proteomic analysis of mouse gastrocnemius muscle revealed that there are 33 up-regulated proteins and 20 down-regulated proteins in elderly muscles compared with young ones [10]. A proteomic study of muscle aging in postmenopausal women found three different regulatory proteins: CA2, MYL2 and SELENBP1 [11]. Ca2 plays a crucial role in the process of metabolism, removing and supplying carbon dioxide, and participates in the regulation of acid-base equilibrium and Ion homeostasis [12,13,14]. Myl2 is a component of myosin in skeletal muscle and is thought to have a modulatory effect on muscle contraction [15,16,17], and SELENBP1 is known to regulate ion homeostasis [11] and participate in intra-Golgi protein transportation [18] and ubiquitination-mediated protein degradation [19,20].

CA2, MYL2a, MYL2b and SELENBP1 are highly conserved during the evolution of vertebrates. These genes in zebrafish and mammals have similar sequences and specific domains (and active sites) and 3D structures. Li Hongyan reported the expression patterns of ca2, myl2a, myl2b and selenbp1 in the early development stage of zebrafish, the characterization of zebrafish genes ca2, myl2 and selenbp1 and the expression profiles related to time, space and age, indicating that the expression profiles of these genes were remodeled along with age increase, indicating that CA2, MYL2a, MYL2b and SELENBP1 can be used as biomarkers of aging [9].

p53 and p21 are marker genes of DNA damage, which activate the apoptosis pathway after DNA damage and cause cell apoptosis and biological aging [21]. p53 is a stress protein that is rapidly up-regulated after cell stimulation, activating the p21 gene, thus inhibiting the activity of cyclinA/ECDK2 and preventing the phosphorylation of RB and cell cycle progression. p53/p21 and various pathways of cellular senescence induced by oxidative stress involve the changes of p53/p21 gene expression [22]. When H_2_O_2_ was used as an inducer, H_2_O_2_ could induce DNA damage and activate the p53/p21 signal pathway through oxidative stress. Thus, researchers used markers of DNA damage, such as γ-H2AX and p21, as markers of aging [23,24].

*Salvia miltiorrhiza* (Danshen) has the functions of promoting blood circulation and regulating menstruation, removing blood stasis and pain, cooling blood and eliminating carbuncle, clearing away heart trouble, nourishing blood, tranquilizing the mind, etc. [25].

Salvianolic acid B (Sal B) is a major bioactive compound extracted from Salviae miltiorrhiza. It has important effects on various organs such as the heart, brain, liver, kidney and intestines [26]. It has been shown that Sal B has antioxidant [21,27], anti-inflammatory and anti-fibrotic effects, and inhibits apoptosis [28]. The strong antioxidant effects may be one of the bases of the other pharmacological effects of Sal B [29,30,31]. Sal B also exhibited a protective effect against cerebral ischemia-reperfusion injury by the up-regulation of anti-apoptotic protein Bcl-2 expression and maintenance of mitochondrial membrane potential [32]. Zheng Zhiwei demonstrated that Sal B exhibited significant effect on protection against aminoglycoside antibiotics and cisplatin-induced ototoxicity by suppressing the production of ROS and mitochondrial apoptotic pathway, indicating that Sal B could be a preventive or therapeutic agent in the treatment of ototoxic insult-induced hearing loss [33]. Florence Hui Ping Tan also demonstrated that Danshen water extract (DWE) and its components (Sal A and Sal B) may have therapeutic potential for AD patients and possibly other forms of brain diseases [34]. However, the result and mechanism of action of Sal B on cell and embryo aging has not been clearly expounded.

This study’s aim was to establish the models for accelerating the senescence of zebrafish embryos, and analyze the molecular mechanism of promoting aging, and then study whether Sal B possesses a protective action against ethanol-induced aging in zebrafish embryo and, if so, the possible mechanisms underlying this action.

In this research, we first organized an environmental toxicity test for 120 h after zebrafish embryo fertilization, observing and calculating the mortality rate, malformation rate and fertilization rate, in order to determine the toxicity of the reagents and the 96 hpf semi-lethal dose (which means the concentration of reagent leading to the death of half of all embryos) to carry out the following molecular experiments. Then, we treated the embryos with the selected 96 hpf semi-lethal dose for 6 h, extracted the mRNA for qRT-PCR to test the aging and oxidative stress-related genes and tested the activation of oxidative related enzymes.

## 2. Results

### 2.1. Ethanol Aging Model Establishment

**Figure 1:**
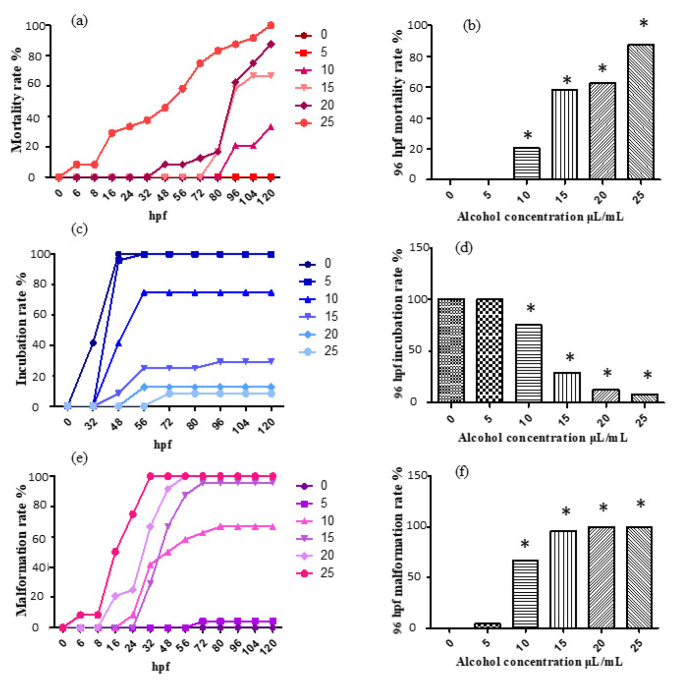
The mortality rate, incubation rate and malformation rate after ethanol treatment. The results were analyzed by χ^2^ test, vs. the control group (0 μL/mL ethanol): * *p* < 0.05. (**a**) mortality rate; (**b**) mortality rate at 96 hpf; (**c**) incubation rate; (**d**) incubation rate at 96 hpf; (**e**) malformation rate; (**f**) malformation rate at 96 hpf.

For the treatment with 0, 5, 10, 15, 20 and 25 μL/mL ethanol solution, the mortality rate, incubation rate and malformation rate varying with time are shown, respectively, in Figure 1a,c,e. The mortality rate, incubation rate and malformation rate at 96 h past fertilization are shown, respectively, in Figure 1b,d,f.

According to the results, the 5 μL/mL experimental group was considered to show no obvious changes, as it did not show much difference compared with the control; the 10 μL/mL, 15 μL/mL, 20 μL/mL and 25 μL/mL experimental groups exhibited significant differences compared with the control. Moreover, the mortality rate and malformation rate both increased evidently, and the incubation rate showed an obvious trend of decrease. The results exhibit an evident effect of ethanol induction, indicating the capability of ethanol aging model establishment. The 96 hpf median lethal dose was approximately 15 μL/mL.

**Figure 2:**
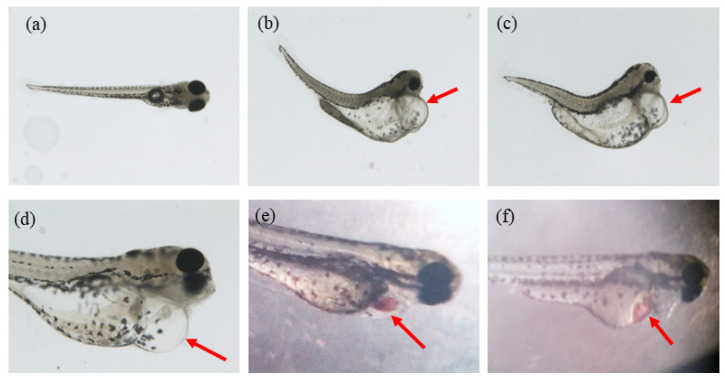
The various malformation after being ethanol-treated. (**a**) normal; (**b**,**c**) pericardial edema, yolk sac deformity and spinal curvature; (**d**) pericardial edema; (**e**) heart bleeding; (**f**) liver bleeding.

According to the observations during the experiment, the ethanol treatment has an obvious effect on morphological changes. It was noticed that malformations of different extents and kinds appeared in the experimental groups. The various malformation included spinal curvature, heart bleeding, liver bleeding, yolk sac deformity, pericardial edema of different extents (usually accompanied by small heart), etc. (Figure 2a–f).

### 2.2. H_2_O_2_ Aging Model Establishment

**Figure 3:**
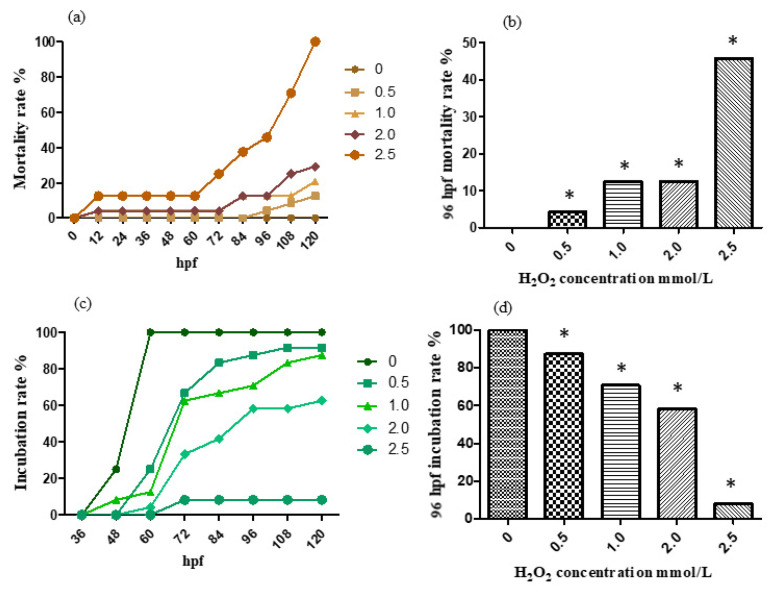
The mortality rate (**a**,**b**) and incubation rate (**c**,**d**) after being H_2_O_2_-treated. The results were analyzed by χ^2^ test, vs. the control group (0 μL/mL ethanol): * *p* < 0.05.

For the treatment with 0, 0.5, 1.0, 2.0 and 2.5 mmol/L H_2_O_2_ solution, the mortality rate and incubation rate varying with time are shown, respectively, in Figure 3a,c. The mortality rate and incubation rate at 96 h past fertilization are shown, respectively, in Figure 3b,d. The hydrogen peroxide model has a significant effect on development. The zebrafish embryos in the treatment group are generally delayed in hatching with swollen abdomens and the suspect of liver damage. Moreover, the high concentration group died about one day after incubation, indicating that the egg membrane has blocked the effect of hydrogen peroxide on the embryo to some extent.

According to the results, the mortality rate preformed a trend of increase with concentration addition in general. The 96 hpf median lethal dose was approximately 2.5 mmol/L.

### 2.3. 6-OHDA Aging Model Establishment

**Figure 4:**
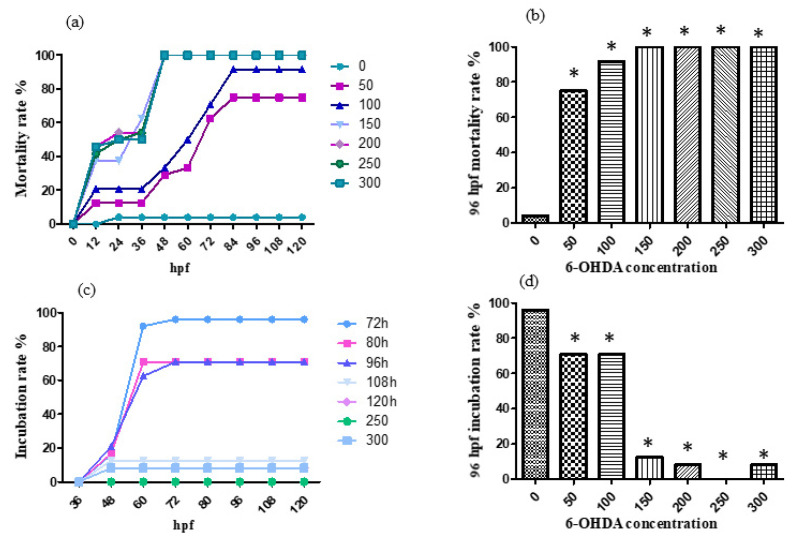
The mortality rate (**a**,**b**) and incubation rate (**c**,**d**) after being 6-OHDA-treated (at a high concentration). The results were analyzed by χ^2^ test, vs. the control group (0 μL/mL ethanol): * *p* < 0.05.

For the treatment with 0, 50, 100, 150, 200, 250 and 300 μmol/L 6-OHDA solution, the mortality rate and incubation rate varying with time are shown, respectively, in Figure 4a,c. The mortality rate and incubation rate at 96 h after fertilization are shown, respectively, in Figure 4b,d. 6-OHDA has strong neurotoxicity and an obvious effect on the development of zebrafish nerves. Zebrafish in the treatment group generally have slight head enlargement, and embryos often die before hatching.

**Figure 5:**
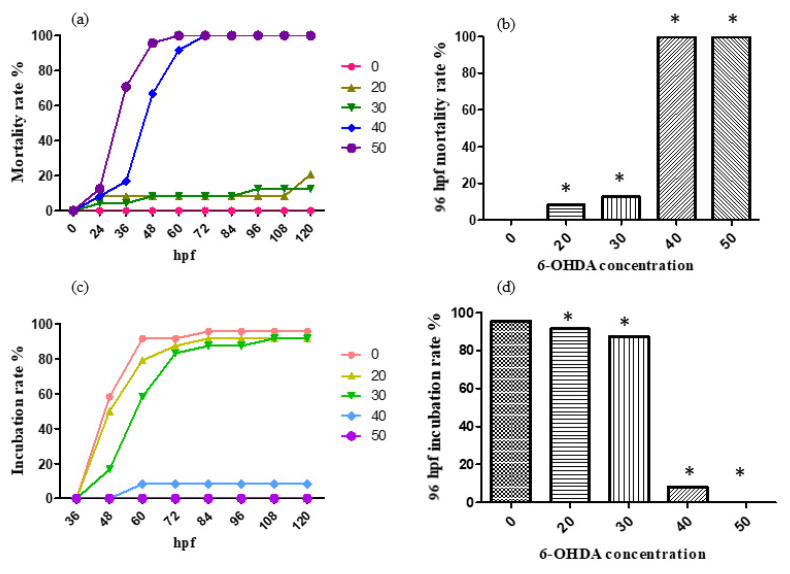
The mortality rate (**a**,**b**)and incubation rate (**c**,**d**) after being 6-OHDA-treated (at a low concentration, 0–50 μmol/L). * *p* < 0.05.

According to the results, the designed concentrations of 6-OHDA were too high, so the concentrations were adjusted to 0, 10, 20, 30, 40 and 50 μmol/L. For the treatment with 0, 10, 20, 30, 40 and 50 μmol/L 6-OHDA solution, the mortality rate and incubation rate varying with time are shown, respectively, in Figure 5a,c. The mortality rate and incubation rate at 96 h past fertilization are shown, respectively, in Figure 5b,d.

According to the results, the 96 hpf median lethal dose was approximately 30 μmol/L.

### 2.4. Expression of Aging-Related Genes Up-Regulated after Aging Induction

**Figure 6:**
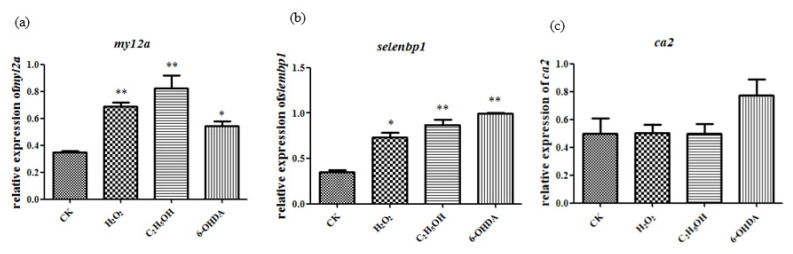
Ethanol, H_2_O_2_ and 6-OHDA promote the expression of *myl2a* (**a**), *selenbp1* (**b**) and *ca2* (**c**). The statistical results shown represent the means (*n* = 3); vs. the control group (*n* = 3) * *p* < 0.05, ** *p* < 0.01.

The expression of aging-related genes *ca2*, *myl2a* and *selenbp1* were detected following the method of Maiquan Li [2]. The results showed that *myl2a* and *selenbp1* had significant changes compared to control group after ethanol, H_2_O_2_ and 6-OHDA treatment, while *ca2* expression did not show significant changes (Figure 6). Thus, in the following analysis, *myl2a* and *selenbp1* were chosen as molecular markers.

### 2.5. Expression of Nrf2 and Sod Genes Were Also Up-Regulated after Aging Induction

**Figure 7:**
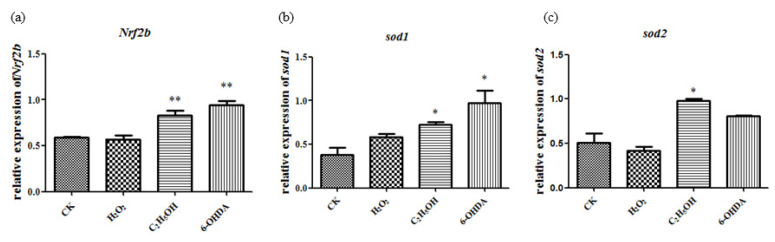
Ethanol, H_2_O_2_ and 6-OHDA promote the expression of *Nrf2b* (**a**), *sod1* (**b**) and *sod2* (**c**). The statistical results shown represent the means (*n* = 3); vs. the control group * *p* < 0.05, ** *p* < 0.01.

The expression level of *sod1*, *sod2* and *Nrf2b* show significant changes compared to the control group after ethanol, H_2_O_2_ and 6-OHDA treatment under the concentration of 15 μL/mL, 2.5 mmol/L and 30 μmol/L, respectively (Figure 7).

Simultaneous changes of aging-related genes and oxidative stress-related genes in gene expression suggest that the aging models established by ethanol, H_2_O_2_ and 6-OHDA are associated with oxidative stress.

### 2.6. Ethanol-Induced Damage and Aging Recovered after Salvanic Acid B Treatment

#### 2.6.1. Embryo Mortality Reduced after Sal B Treatment

The embryonic death rate after ethanol treatment was 33.8%, and it decreased to 24% with a low-concentration (0.05 μg/mL) Sal B treatment and 17.6% with a high-concentration (0.5 μg/mL) Sal B treatment. This means that Sal B can protect against ethanol. When the concentration of Sal B increased to 4 μg/mL, all the embryos died, which indicated that a higher concentration of Sal B was harmful to fish embryos. Sal B should be used to account for the concentration range.

#### 2.6.2. Oxidative Stress Decreased after Sal B Treatment

**Figure 8:**
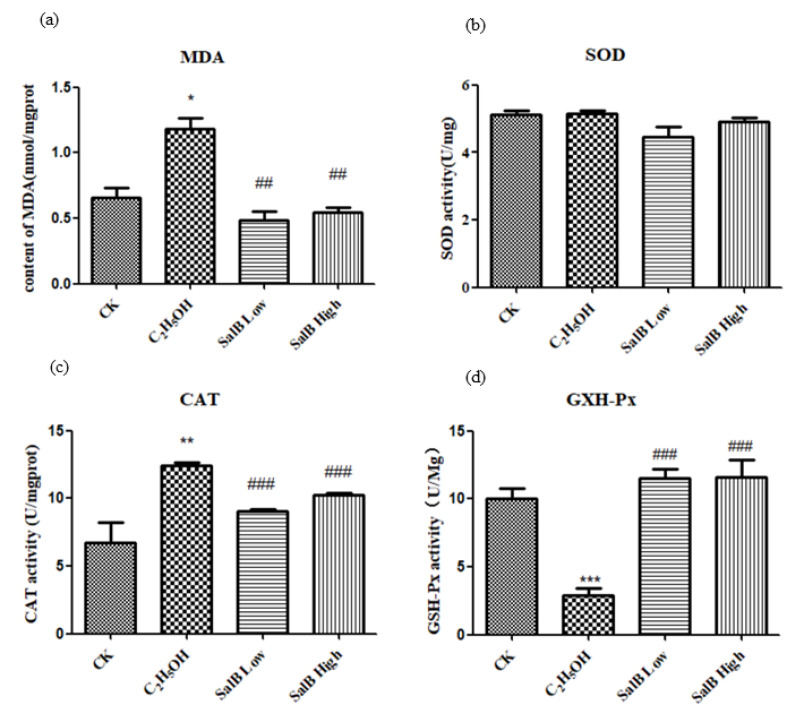
Salvianolic acid can inhibit oxidative stress caused by ethanol. (**a**) The content changes of MDA increased after adding ethanol; (**b**) SOD activity did not change; (**c**) CAT activity increased; (**d**) GXH-Px activity decreased. The statistical results shown represent the means (*n* = 3); vs. the control group (*n* = 3) * *p* < 0.05, ** *p* < 0.01. vs. the ethanol-treated group # *p* < 0.05, ## *p* < 0.01, ### *p* < 0.001.

The level of MDA was used to reflect the oxidative stress. The content of MDA increased after adding ethanol, and decreased or returned to normal after adding Sal B (Figure 8a).

#### 2.6.3. Antioxidant Enzyme Activity Changed after Sal B Treatment

After ethanol treatment, SOD activity did not change significantly (Figure 8b), while CAT activity increased significantly (Figure 8c) and GXH-Px activity decreased significantly (Figure 8d). It is suggested that different enzyme activities have different sensitivities to ethanol.

After adding Sal B, the activity of SOD, CAT and GXH-Px were restored to the control level, which suggested that the regulation of SOD, CAT and GXH-Px by Sal B might be different.

#### 2.6.4. Antioxidant-Related Gene Expression Changed after Sal B Treatment

**Figure 9:**
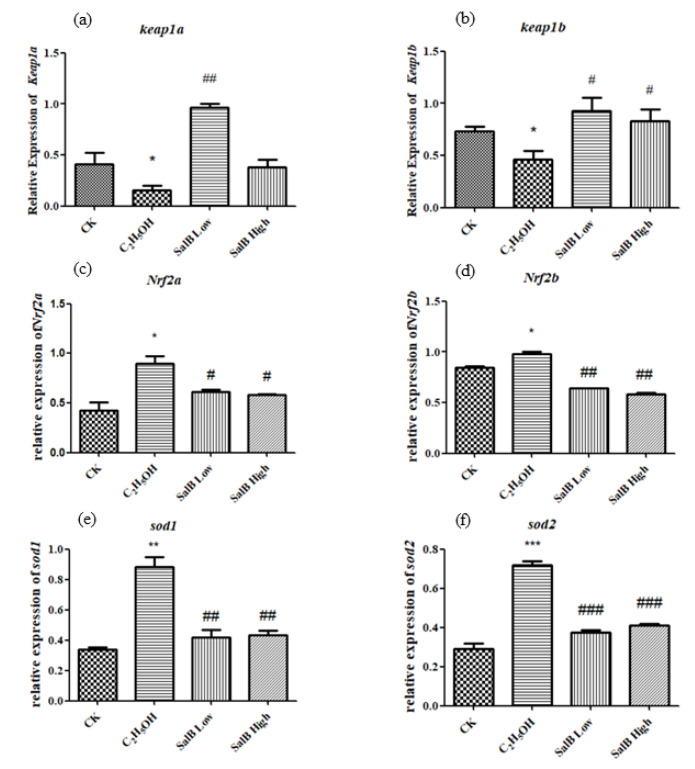
The expression of *keap1a* (**a**), *keap1b* (**b**), *Nrf2a* (**c**), *Nrf2b* (**d**), *sod1* (**e**) and *sod2* (**f**) after Sal B was added in the group of ethanol acute toxicity treatment. The statistical results shown represent the means (*n* = 3); vs. the control group (*n* = 3) * *p* < 0.05, ** *p* < 0.01, *** *p* < 0.001 vs. the ethanol-treated group # *p* < 0.05, ## *p* < 0.01, ### *p* < 0.001.

The expressions of *keap1a* and *keap1b* were down-regulated after adding ethanol, and showed significant difference compared with control group (Figure 9b). The expressions of *Nrf2a*, *Nrf2b*, *sod1* and *sod2* were up-regulated after adding ethanol, and showed significant difference with control group (Figure 9c–f). After adding Sal B, the expression of *keap1a* and *keap1b* were up-regulated, while *Nrf2a*, *Nrf2b*, *sod1* and *sod2* were down-regulated and returned to the control state. This suggests that Sal B have the effect of recovering the oxidative damage caused by ethanol through regulating the expression of the keap1/Nrf2 pathway and antioxidant enzymes downstream.

#### 2.6.5. Aging-Related Genes *myl2a*, *selenbp1*, *p53* and *p21* Expression Decreased after Salvanic Acid B Treatment

**Figure 10:**
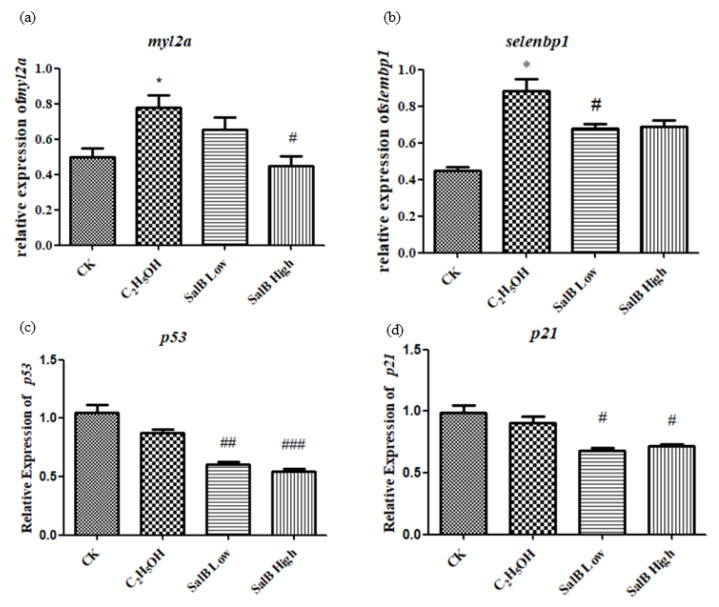
The expression of *myl2a* (**a**), *selenbp1* (**b**), *p53* (**c**) and *p21* (**d**) after Sal B added in the group of ethanol acute toxicity treatment. The statistical results shown represent the means (*n* = 3); vs. the control group (*n* = 3) * *p* < 0.05, ** *p* < 0.01, *** *p* < 0.001 vs. the ethanol-treated group (*n* = 3) # *p* < 0.05, ## *p* < 0.01, ### *p* < 0.001.

The expression of aging-related genes *myl2a* and *selenbp1* was up-regulated after adding ethanol, and showed a significant difference compared with the control group (Figure 10a,b). After adding Sal B, the expression of *myl2a* and *selenbp1* were down-regulated. This suggests that Sal B have the effect of recovering the aging caused by ethanol. The expressions of *p53* and *p21* induced by apoptosis were also examined. The results showed that ethanol did not up-regulate the expression of *p53* and *p21*. However, when Sal B was added, the expression of *p53* and *p21* decreased (Figure 10c,d).

## 3. Discussion

The advantages of zebrafish in morphology, development, genetic similarity, etc. make it an important and widely used model organism for studying vertebrate development mechanisms [35,36]. Zebrafish have many similar aging characteristics to mammals [37]. Therefore, they have also become an ideal object for studying aging and rising model organism for aging research.

The results show that the morphological changes of the three-drug treatment established aging models are slightly different. However, gene expression tests showed that oxidative stress- and aging-related genes were significantly up-regulated, indicating that the modeling was basically successful. The aging model was established through the oxidative stress pathway. While leading to embryo malformation, ethanol (C_2_H_5_OH), hydrogen peroxide (H_2_O_2_) and 6-hydroxydopamine (6-OHDA) induced the expression of senescence marker genes in zebrafish, and further accelerated the aging process.

Based on the research of Li Hongyan, we used these three genes as indicators to detect gene expression changes after treatment with hydrogen peroxide, ethanol and 6-OHDA in this study. Ca2 did not change significantly, while MYL2b and SELENBP1 significantly increased after induction.

There was no significant difference between p53 and p21 expression induced by ethanol, which suggested that ethanol did not induce DNA damage.. However, p53 and p21 decreased significantly after Sal B was added, suggesting that Sal B could down-regulate p53 and p21, and then can decrease the occurrence of apoptosis, and presumably reduce the aging process induced by DNA damage.

At the same time, the expression of oxidative stress-related genes was detected, and it was found that the genes related to oxidative stress were significantly up-regulated after induction. Therefore, it is speculated that this aging model is related to the generation of reactive oxygen species, which is consistent with the theory of aging free radicals.

Sal B is a polyphenol and its molecular structure contains many covalent bonds, most of which are sp2 hybridized and form a large p-π conjugated system [31,38]. These electron-deficient centers can often make the molecule oxidation-resistant. When zebrafish embryos are subjected to oxidative stress, the parasite phenolic group of Sal B is oxidized to the corresponding oxime, which can bind with Keap1, resulting in a conformational change to Keap1 and release of Nrf2, thus inducing a cascade of downstream gene expression [30,31]. While Sal B has a Danshensu structure, it can promote Nrf2 expression [39] and reduce ROS production [18]. Based on its effect in anti-oxidation, Sal B could be used in protection against aging through antioxidants, which broadened its application. The reported molecules that have neuroprotective and antioxidant actions include anacardic acid (AA). According to previous research, AA exhibits significant anticonvulsant and antioxidant activities in S. cerevisiae mutated for superoxide dismutases (SOD) [40].

In our experiment, ethanol and Sal B were added together. Since Sal B can remove oxygen radicals by providing hydrogen atoms, it is assumed that the removal of oxidative components from the environment can be accomplished in solution to reduce oxidative stress. Moreover, the expression of sod could be regulated by keap1/Nrf2 pathway, which could also reduce the level of oxidative stress.

We also used the bioinformatics method to predict the proteins and genes involved in this study. The significantly enriched cellular components (CC) of oxidative-related genes in zebrafish are mainly located in the intracellular and membranous regions, including those listed in Table S1, such as the inclusion body, cytosolic ribosome, ribosome, cytosol, mitochondrion, cytoplasm, intracellular, membrane, intrinsic component of membrane and integral component of membrane.

Obviously enriched biological processes (BP) are mainly present in the cellular metabolic processes and cellular response to stress. Table S1 listed the 59 processes, such as the glutamate catabolic process, superoxide metabolic process, hydrogen peroxide metabolic process, superoxides, toxic substances, oxidative stress, xenobiotics, etc.

The molecular function of the related genes enriched 19 meaningful items, mainly redox-related enzyme activities, such as glutamate dehydrogenase [NAD(P)+] activity, oxidoreductase activity, superoxide dismutase activity, peroxiredoxin activity, antioxidant activity, catalytic activity, etc.

The KEGG signal pathway of oxidative stress-related genes in zebrafish was enriched and analyzed. The results of 23 meaningful pathways are shown in Table S2.

The selected KEGG signal pathways are mainly metabolic pathways and necroptosis-related signal pathways, such as glutathione metabolism, metabolic pathways, peroxisome, citrate cycle (TCA cycle), necroptosis, ferroptosis, autophagy, etc.

**Figure 11:**
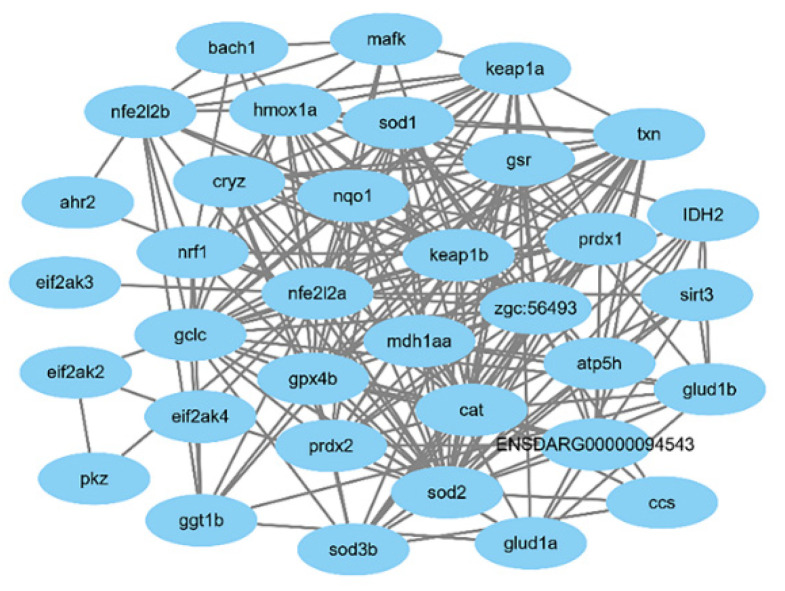
The protein–protein interactions of oxidative-related genes.

In this study, the protein–protein interaction (PPI) network of the identified oxidative stress-related genes *Nrf2a*, *Nrf2b*, *sod1* and *sod2* in zebrafish was constructed through the STRING database, as shown in Figure 11.

According to the bioinformatics analysis, Sal B could probably respond to aging damage by regulating many more genes, and it can also protect against other damages caused by oxidative stress.

## 4. Materials and Methods

### 4.1. Zebrafish Culture

The wild-type AB strain zebrafish (*Danio rerio*) used in this study were from the Heilongjiang River Fishery Research Institute of Chinese Academy of Fishery Sciences (HRFIR) (Heilongjiang, China). Before the experiments, all the zebrafish were kept in aerated circulating water for more than two weeks at 28 ℃ (water temperature) with a light/dark cycle of 14 h/10 h. One day before the start of the experiment, healthy zebrafish were selected and placed in a zebrafish mating box at a female to male ratio of 1:2, and the female and male fish were separated by a transparent baffle. On the day of the experiment, the baffle was removed at around 10 am, allowing the zebrafish to mate and lay eggs naturally. Then, fish eggs were collected after one or two hours.

### 4.2. Establishment of Aging Models

Ethanol (C_2_H_5_OH), hydrogen peroxide (H_2_O_2_) and 6-hydroxydopamine (6-OHDA) were chosen as aging induction reagents in the experiments. All the three aging induction reagents were dissolved into zebrafish breeding E3 solution.

Healthy zebrafish embryos were collected after microscope observation. Then, they were exposed to aging-inducing solutions in 6- or 24-well cell culture plates at 4 to 6 h after fertilization.

The gradient concentration of ethanol solution was 5, 10, 15, 20 and 25 μL/mL. The gradient concentration of hydrogen peroxide solution was 0.5, 1.0, 2.0 and 2.5 mmol/L. The gradient concentration of 6-hydrosydopamine solution was 20, 30, 40, 50, 100, 150, 200, 250 and 300 μmol/L. The three experimental groups were each accompanied with zebrafish bred with E3 water as the control group.

During the experiment, the embryo breeding water of each group was replaced every 24 h, and the dead individuals were selected to prevent normal embryos from being affected by the dead ones.

Then, the zebrafish were observed twice a day at regular times for 120 h after fertilization. In the ethanol experimental group, the zebrafish were observed at 6, 8, 16, 24, 32, 48, 56, 72, 80, 96, 104 and 120 hpf (hours past fertilization). In the hydrogen peroxide and 6-hydrosydopamine experimental groups, the zebrafish were observed at 12, 24, 36, 48, 60, 72, 84, 96, 108 and 120 hpf (hours past fertilization). During the observations, the mortality, state of malformation and incubation was recorded and the mortality rate, malformation rate and incubation rate were calculated.

### 4.3. Morphological Observation

After the observation of the aging models at 120 hpf, several examples from each experimental group were selected. These zebrafish were washed with double distilled water, fixed by paraformaldehyde and then photographed under a dissecting microscope(Agilent Technologies Inc, Santa Clara, CA, USA).

### 4.4. Significant Concentration of Aging Agent Treatment 

After 6 h of fertilization, zebrafish embryos were exposed to a 96 hpf semi-lethal dose for 6 h. The treated dose of ethanol, H_2_O_2_ and 6-OHDA solvent was 15 μL/mL, 2.5 mmol/L and 30 μmol/L, respectively. Then, the embryos were collected for mRNA extraction and real-time PCR.

### 4.5. Sal-B Treatment

Sal B was added to the acute toxicity test at the same time. The concentration of Sal B was 0.05 μg/mL in the low dose group, and 0.5 μg/mL in the high dose group. Then, the embryos were collected for mRNA extraction and real-time PCR.

### 4.6. Antioxidant Enzyme Activity Detection

The content of MDA in zebrafish embryos and the activities of SOD, CAT and GSH-xP were measured using Malondialdehyde (MDA) assay kits, Superoxide Dismutase (SOD) assay kits, Catalase (CAT) assay kits and Glutathione Peroxidase (GSH-Px) assay kits. These kits were all purchased from the Nanjing Jiancheng Bioengineering Institute, China. The zebrafish embryo protein was detected according to the manual of the BCA protein assays kit (Nanjing Jiancheng Bioengineering Institute). The content of MDA and the activity of SOD, CAT and GSH-Px was calculated by the following formula. The content of MDA and the activity of SOD, CAT and GSH-Px = the content of MDA and the activity of SOD, CAT and GSH-Px in the sample/total protein in sample.

### 4.7. Total RNA Extraction and Real-Time PCR

Total RNA was extracted using Trizol. After the RNA was obtained, absorbance was measured at 260 and 280 nm to determine the concentration and purity. Then, 1 µg of total RNA was used for reverse transcription..

The concentration of RNA was determined and the reverse transcription test was carried out in time. The reverse transcription of RNA was carried out in two steps using a reverse transcription kit (Takara, Takara, Beijing, China), namely genomic DNA removal and reverse transcription. After the reaction, the samples were stored at −20 ℃ for a long time.

The ABI 7500 sequence detection system was used (Applied Biosystem) for real-time PCR detection. The sequences of the PCR primers used are shown in Table 1.

TaqMan cycle number (Ct) was normalized using the 2∆∆Ct method. The relative expression rate (∆Ct) of the target gene/internal control gene of each sample was calculated first, after which the mean value of ∆Ct in the control group was calculated, while the ∆∆Ct (relative expression rate of the treated group/control group) is obtained by subtracting the ∆Ct value of the control group from each ∆Ct value of the treated group. The expression quantity of each sample in the treated group was calculated by the formula 2-∆∆Ct, after which the average value of the treated group was calculated.

### 4.8. Data Analysis

All the experiments were repeated at least three times. Statistical significance was performed using student’s *t*-test or χ^2^ test. Differences were considered to be significant at *p* < 0.05.

### 4.9. Bioinformatics Analysis

Functional enrichment analysis of differentially expressed genes were carried out through the GO database [http://geneontology.org/ (accessed on 10 October 2020)] the biological processes (BP), molecular function (MF) and cellular component (CC) in which genes are involved are analyzed. Pathway enrichment analysis were carried out through KEGG database [https://www.kegg.jp/ (accessed on 10 October 2020)], the KEGG signal pathway in which the differentially expressed genes are mainly involved was obtained when *p* < 0.05 was used as the significance threshold. Using STRING database [https://string-db.org/ (accessed on 11 October 2020)] and Cytoscape [https://cytoscape.org/ (accessed on 11 October 2020)]to study the protein–protein interaction network encoded by differentially expressed genes.

### 4.10. Ethics Statements

This study was carried out in strict accordance with the recommendations of the Guide for the Care and Use of Laboratory Animals of Chinese society of experimental animals and the National Institutes of Health guide for the care and use of Laboratory animals (NIH Publications No. 8023, revised 1978). The protocol was approved by the ethical committee of the Heilongjiang Medical and Laboratorial Animal Center. The approval number of the ethics committee that endorsed this study is 2019022.

## 5. Conclusions

After the zebrafish embryos were treated with ethanol, H_2_O_2_ and 6-OHDA, the mortality rate, malformation rate and incubation rate increased, while the aging-related genes *myl2a* and *selenbp1* were significantly up-regulated compared to control group. The results showed that the aging model was successfully established, and the effect of ethanol was remarkable. In addition, the expression of aging-related genes and oxidative stress-related genes *Nrf2b*, *sod1* and *sod2* were also up-regulated, indicating that the model was associated with oxidative stress. After treatment with Sal B, the oxidative stress induced by ethanol was down-regulated, and the expression of aging-related genes were down-regulated. The bioinformatics analysis also showed that oxidative stress-related factors were associated with a variety of cellular functions and physiological pathways, and therefore, we can predict that Sal B could protect against a variety of oxidative stress-related injuries, including aging. This conclusion has further widened the range of usage of Sal B.

## Figures and Tables

**Table 1:** Real-time PCR primers.

Gene Name	Primer Sequence
Elfa	F: 5’-GGCCCCTGCCAATGTA-3’ R: 5’-GGGCTTGCCAGGGAC-3’
Nrf2a	F: 5’-GAGCGGGAGAAATCACACAGAATG-3’ R: 5’-CAGGAGCTGCATGCACTCATCG-3’
Nrf2b	F: 5’-GCCACGTTATGCTGGGTTTC-3’ R: 5’-CTGCGGACAACGATAGCAGA-3’
sod1	F: 5’-CTAGCCCGCTGACATTACATC-3’ R: 5’-TTGCCCACATAGAAATGCAC-3’
sod2	F: 5’-CGCATGTTCCCAGACATCTA-3’ R: 5’-GAGCGGAAGATTGAGGATTG-3’
myl2a	F: 5’-AAAAGGCCGTCCATCCCATT-3’ R: 5’-TTTTTAGGAGCCATGCCGGA-3’
ca2	F: 5’-CAGACGACAAGGGCTCCGAAC-3’ R: 5’-AAAACCCCAACCACAGCAAGA-3’
selenbp1	F: 5’-CGAGGTGGTAATCGACATTTG-3’ R: 5’-AATAACGCGGCGAAGAAGATG-3’
keap1a	F: 5’-GTGTGGAGTGCTACTGTCCC-3’ R: 5’-TCCTCCTCTGGCAGGATACC--3’
keap1b	F: 5’-ATCGAGGGGATACACCCCAA-3’ R: 5’-AGCTGCTCCACCAGGAAATC-3’
p21	F: 5’-CACGCCCACAGCACACCATATC-3’ R: 5’-CGGTCATCTTTCCCTTGCCCTTC-3’
p53	F: 5’-GATGTGGTGCCTGCCTCAGATG-3’ R: 5’-GAGTCGCTTCTTCCTTCGTCCTTC-3’

## Data Availability

All data are available in this publication and in the Supplementary Materials.

## Supplementary Materials

The following are available online, Table S1: GO functional enrichment analysis for the oxidative related-genes, Table S2: KEGG pathway enrichment analysis for the oxidative related-genes.

## Sample Availability

Samples of the compounds are available from the authors.
