# Supplementary material for: Protective Effects of Sal B on Oxidative Stress-Induced Aging by Regulating the Keap1/Nrf2 Signaling Pathway in Zebrafish
Source: Molecules. 2021 Aug 29;26(17):5239. doi: 10.3390/molecules26175239 (PMC8434535; doi:10.3390/molecules26175239)
Supplement: Supplementary file 1 [file molecules-26-05239-s001.zip › molecules-1357934-supplementary.pdf]

Table S1 GO functional enrichment analysis for the oxidative related-genes

category	term	count	P-value	Fold enrichment	FDR
**GOTERM_CC _DIRECT**	inclusion body	2	3.09E-05	> 100	8.50E-03
	cytosolic ribosome	4	5.40E-06	35.96	2.47E-03
	ribosome	4	9.66E-05	16.85	1.66E-02
	cytosol	11	4.81E-08	8.09	6.61E-05
	mitochondrion	8	2.72E-05	6.33	9.35E-03
	cytoplasm	24	6.94E-08	2.63	4.77E-05
	intracellular	26	4.93E-05	1.78	1.13E-02
	membrane	1	8.71E-05	0.1	1.71E-02
	intrinsic component of membrane	0	2.47E-04	< 0.01	3.40E-02
	integral component of membrane	0	2.44E-04	< 0.01	3.73E-02
**GOTERM_BP _DIRECT**	glutamate catabolic process	2	8.85E-06	> 100	1.91E-03
	removal of superoxide radicals	6	3.67E-15	> 100	8.54E-12
	cellular response to superoxide	6	3.67E-15	> 100	6.83E-12
	cellular response to oxygen radical	6	3.67E-15	> 100	5.69E-12
	cellular oxidant detoxification	6	3.67E-15	> 100	4.88E-12
	response to oxygen radical	6	3.67E-15	> 100	4.27E-12
	response to superoxide	6	3.67E-15	> 100	3.79E-12
	cellular detoxification	6	1.58E-13	> 100	1.05E-10
	cellular response to prostaglandin stimulus	3	2.80E-07	> 100	8.14E-05
	cellular response to reactive oxygen species	6	1.58E-13	> 100	9.82E-11
	cellular response to toxic substance	6	1.58E-13	> 100	9.21E-11
	cellular response to fatty acid	3	3.73E-07	> 100	1.05E-04
	response to prostaglandin	3	4.85E-07	> 100	1.29E-04
	ether metabolic process	2	5.29E-05	> 100	1.00E-02
	glycerol ether metabolic process	2	5.29E-05	> 100	9.83E-03
	detoxification	6	6.26E-13	> 100	3.23E-10
	cellular response to oxidative stress	11	5.28E-23	> 100	2.45E-19
	response to reactive oxygen species	7	9.03E-15	> 100	7.63E-12
	response to toxic substance	7	1.14E-14	> 100	8.80E-12
	cellular response to chemical stress	11	1.35E-22	> 100	4.18E-19
	dicarboxylic acid catabolic process	2	6.60E-05	> 100	1.18E-02
	superoxide metabolic process	6	1.25E-12	> 100	5.55E-10
	response to fatty acid	3	9.47E-07	> 100	2.38E-04
	response to methylmercury	2	9.67E-05	> 100	1.70E-02
	response to oxidative stress	15	8.05E-29	> 100	7.49E-25
	glutamate metabolic process	3	1.92E-06	> 100	4.70E-04
	hydrogen peroxide catabolic process	3	3.86E-06	> 100	8.97E-04

	reactive oxygen species metabolic process	7	7.05E-13	> 100	3.28E-10
	hydrogen peroxide metabolic process	3	4.90E-06	> 100	1.11E-03
	cell redox homeostasis	6	1.39E-10	85.16	4.98E-08
	dicarboxylic acid metabolic process	4	3.90E-07	71.91	1.07E-04
	glutathione metabolic process	3	1.64E-05	65.59	3.38E-03
	response to xenobiotic stimulus	7	2.77E-11	62.23	1.03E-08
	cellular response to xenobiotic stimulus	6	1.25E-09	57.79	4.00E-07
	response to inorganic substance	7	1.65E-10	47.59	5.68E-08
	glutamine family amino acid metabolic process	3	4.78E-05	44.94	9.26E-03
	cellular response to lipid	3	1.42E-04	30.72	2.40E-02
	cellular response to oxygen-containing compound	9	2.60E-11	28.22	1.01E-08
	response to metal ion	3	2.47E-04	25.28	3.97E-02
	response to oxygen-containing compound	11	6.39E-13	23.86	3.13E-10
	response to lipid	4	5.31E-05	19.73	9.69E-03
	oxidation-reduction process	15	4.05E-15	16.01	3.77E-12
	cellular response to stress	13	2.22E-12	14.08	9.40E-10
	response to hormone	4	2.14E-04	13.65	3.55E-02
	cellular response to chemical stimulus	14	2.71E-12	11.75	1.09E-09
	cellular homeostasis	7	2.00E-06	11.58	4.78E-04
	response to stress	17	1.29E-13	9.55	9.22E-11
	homeostatic process	10	6.46E-08	9.28	2.00E-05
	response to chemical	17	2.28E-13	9.22	1.25E-10
	response to organic substance	8	1.19E-05	7.1	2.52E-03
	cellular catabolic process	9	6.55E-06	6.45	1.45E-03
	organonitrogen compound catabolic process	6	3.00E-04	6.41	4.74E-02
	catabolic process	9	2.12E-05	5.57	4.28E-03
	regulation of biological quality	10	1.15E-04	3.99	1.98E-02
	metabolic process	26	3.22E-10	2.98	1.07E-07
	cellular metabolic process	22	8.65E-08	2.91	2.60E-05
	response to stimulus	20	8.20E-07	2.87	2.12E-04
	cellular response to stimulus	16	3.59E-05	2.79	7.10E-03
	cellular process	28	2.36E-04	1.56	3.86E-02
**GOTERM_MF_DIRECT**	glutamate dehydrogenase [NAD(P)+] activity	2	8.85E-06	> 100	3.31E-03
	glutamate dehydrogenase (NAD+) activity	2	8.85E-06	> 100	2.98E-03

	oxidoreductase activity, acting on the CH-NH2 group of donors, NAD or NADP as acceptor	2	8.85E-06	> 100	2.71E-03
	eukaryotic translation initiation factor 2alpha kinase activity	4	2.40E-10	> 100	2.70E-07
	oxidoreductase activity, acting on superoxide radicals as acceptor	4	4.00E-10	> 100	3.37E-07
	superoxide dismutase activity	4	4.00E-10	> 100	2.69E-07
	thioredoxin peroxidase activity	2	3.09E-05	> 100	6.94E-03
	peroxiredoxin activity	2	5.29E-05	> 100	1.05E-02
	antioxidant activity	9	7.48E-16	95.8	1.26E-12
	disulfide oxidoreductase activity	3	1.19E-05	73.55	3.34E-03
	peroxidase activity	4	5.37E-07	66.04	2.59E-04
	copper ion binding	3	2.19E-05	59.2	5.67E-03
	oxidoreductase activity, acting on peroxide as acceptor	4	8.30E-07	58.84	3.50E-04
	oxidoreductase activity, acting on a sulfur group of donors	3	4.29E-05	46.67	9.05E-03
	oxidoreductase activity, acting on NAD(P)H	3	1.00E-04	34.67	1.88E-02
	translation regulator activity	4	2.33E-05	24.52	5.60E-03
	oxidoreductase activity	18	4.35E-19	17.63	1.47E-15
	catalytic activity	25	1.09E-09	3.01	6.14E-07

Table S2 KEGG pathway enrichment analysis for the oxidative related-genes

No.	Biological process/pathway	count	P-value
1	Glutathione metabolism	5	7.67E-09
2	Metabolic pathways	12	2.82E-08
3	Peroxisome	5	5.11E-08
4	Carbon metabolism	5	3.55E-07
5	Protein processing in endoplasmic reticulum	5	1.53E-06
6	Ferroptosis	3	1.90E-05
7	D-Glutamine and D-glutamate metabolism	2	2.35E-05
8	Necroptosis	4	4.39E-05
9	Herpes simplex virus 1 infection	4	5.29E-05
10	Nitrogen metabolism	2	0.000232927
11	Arginine biosynthesis	2	0.000387751
12	Citrate cycle (TCA cycle)	2	0.000691759
13	Glyoxylate and dicarboxylate metabolism	2	0.000812
14	Alanine, aspartate and glutamate metabolism	2	0.001128841
15	Cysteine and methionine metabolism	2	0.00160987
16	NOD-like receptor signaling pathway	2	0.012549233
17	Ubiquinone and other terpenoid-quinone biosynthesis	1	0.012842373
18	Autophagy - animal	2	0.01346951
19	FoxO signaling pathway	2	0.014258268
20	Taurine and hypotaurine metabolism	1	0.016027892
21	2-Oxocarboxylic acid metabolism	1	0.022369052
22	Porphyrin and chlorophyll metabolism	1	0.033891747
23	Pyruvate metabolism	1	0.049392486
